# Promises on the go: A field study on keeping one's word

**DOI:** 10.3389/fpsyg.2023.1097239

**Published:** 2023-03-06

**Authors:** Patricia Kanngiesser, Daniil Serko, Jan K. Woike

**Affiliations:** ^1^Faculty of Education and Psychology, Freie Universität Berlin, Berlin, Germany; ^2^School of Psychology, University of Plymouth, Plymouth, United Kingdom; ^3^Max Planck Research Group iSearch, Max Planck Institute for Human Development, Berlin, Germany; ^4^Center for Adaptive Rationality (ARC), Max Planck Institute for Human Development, Berlin, Germany

**Keywords:** promises, commitments, cheating, honesty, behavioral ethics, behavioral change, decision making

## Abstract

Promises are voluntary commitments to perform a future action and are often thought to be powerful levers for behavioral change. Here we studied the effectiveness of promises in two preregistered, incentivized field experiments with German students (*N* = 406) on the premises of a cafeteria. In Experiment 1, the majority of participants (63%) kept their promise to pay back at least half of a € 4-endowment, even though there was no foreseeable cost of breaking the promise, reputational or otherwise. Significantly fewer participants (22%) paid back money in a control group that faced a simple decision to return money or not. In Experiment 2, the majority of participants (54%) kept their promise to add a provided stamp to a postcard and mail it back (anonymously) within a week. We found similar return rates (52%) for a second group for which the word “promise” was omitted from the commitment. Our findings show that participants kept their word outside the laboratory while pursuing everyday activities even when there were no foreseeable negative consequences for breaking them, demonstrating that promises are effective levers for behavioral change.

## 1. Introduction

When we promise, we voluntarily commit ourselves to doing the promised action in the future (Austin, 1975; Rawls, 1999). Some theorists have argued that, in the absence of sanctions or reputational costs, promises are not binding or just cheap talk (Hobbes, 1651/1960; Farrell, 1987). Empirical studies have shown that this suspicion about the fallibility of promises is widely shared. For example, people distrust politicians to keep their election promises, even though most of these promises are usually at least partially fulfilled (Artés, 2011; Naurin, 2014; Thomson et al., 2017). Moreover, people consistently underestimated others' promise keeping by up to 40% in tasks where, in fact, the vast majority of participants kept their word (Woike and Kanngiesser, 2019). Research has also shown that promises and commitments increase, for example, cooperation (Ostrom et al., 1992; Sally, 1995; Bicchieri, 2002; Ellingsen and Johannesson, 2004; Charness and Dufwenberg, 2006; Balliet, 2009; Koessler, 2022) and truth-telling (Bhanot, 2017; Jacquemet et al., 2021; Kanngiesser et al., 2021). Promises thus appear to be powerful devices for boosting beneficial behaviors and fostering behavioral change.

To date, promises have been studied predominantly in one of four variants: (1) as spontaneous communication in social dilemma or trust experiments in the lab (Ostrom et al., 1992; Ellingsen and Johannesson, 2004; Charness and Dufwenberg, 2006), (2) as hypothetical decisions in vignette studies (Mischkowski et al., 2019), (3) as non-incentivized decisions in the lab (e.g., to take part in a survey; Conrads and Reggiani, 2017), and (4) as forced promises (e.g., to respond honestly; Kataria and Winter, 2013; Heyman et al., 2015). However, all variants have some shortcomings. First, participants in social dilemma experiments could primarily use promises to signal their intentions to cooperate, making it difficult to disentangle the effect of promises *per se* from the coordination effect of signaling prosocial intentions (van den Assem et al., 2012; Ismayilov and Potters, 2016; Woike and Kanngiesser, 2019). Second, hypothetical or non-incentivized decisions may not be indicative of people's behavior with monetary stakes. Third, promises are, per definition, *voluntary* commitments (Rawls, 1999), and participants who are forced to promise would have every right to renege on their promise because they did not give their word voluntarily. Moreover, from an ethical point of view, forced promises disrespect individual autonomy and decision making (Hertwig and Grüne-Yanoff, 2017), which raises questions about their suitability as intervention tools.

One approach that may simultaneously overcome each of these shortcomings investigates the effectiveness of voluntary promises under incentivized conditions. A recent study with crowd sourced participants (Amazon's Mechanical Turk) found that most participants kept their voluntary promises even when it was costly for them (Woike and Kanngiesser, 2019). Yet, promise keeping involved relatively little effort and the online setting may not have fully captured the multitude of factors that influence decisions in everyday settings. Incentivized field experiments with voluntary promises can provide such insights.

There is some evidence from field studies that voluntary promises can be effective under real-world conditions. For example, analyses of participants' behavior and communication in a high stake game show (Golden Balls) revealed that voluntary commitments increased cooperation as compared to elicited commitments (Belot et al., 2010).^1^ In a hotel setting, voluntary commitments to behave pro-environmentally (e.g., to re-use towels) were particularly effective if guests made a specific pledge (rather than a more general pledge) and signaled their intentions *via* a pin (Baca-Motes et al., 2013). Overt signals of voluntary commitment also increased office workers' donations to charity in a field study of more than 250 work places (Kessler, 2017). However, some field studies have found strong selection effects: people who voluntarily promised to pay their taxes on time (and entered a lottery if they complied) had already been more likely to pay their taxes on time in the past as compared to people who did not promise (Koessler et al., 2019).

We contribute to this literature by reporting two preregistered field experiments with students in the vicinity of a large university cafeteria (*N* = 406). We used an incentivized paradigm that gave participants a choice between committing or not committing to a course of action (Woike and Kanngiesser, 2019; Kanngiesser et al., 2021). In Experiment 1, participants had a choice between (a) receiving € 1 and doing nothing else and (b) receiving € 4 under the condition that they promised to pay back € 2 in a sealed envelope at the cafeteria's exit (promise condition). They then indicated their chosen action by ticking a box (e.g., “I choose € 4 and I promise that I will pay back € 2.”). Our paradigm minimized potential selection effects as even (and, in fact, especially) participants who had no intention of keeping their word had an incentive to choose the (higher pay-off) promise option. Participants in the control condition of Experiment 1 were offered a choice between receiving € 1 and receiving € 4, but the higher payment involved no promise and simply gave a choice to pay back some, all or none of the money. All monetary incentives in the study were windfall money. In both conditions, the higher payment amount was sufficient to cover the costs of a full student meal at the cafeteria.^2^ We ensured that payback could not be linked to individual participants to minimize any reputational costs for breaking the promise or not returning any money. We predicted higher payback rates in the promise condition compared to the control condition. Specifically, we expected that more people would pay back at least € 2 in the promise than in the control condition and that average payback rates would be higher in the promise than in the control condition.

In Experiment 2, promise keeping required substantially more effort. Moreover, we tested whether removing the word “promise” when asking participants to agree to a course of action would influence their behavior (Woike and Kanngiesser, 2019). It has been argued that promissory obligations arise even without explicitly using the word “promise” as long as the general conditions for promising are fulfilled (Searle, 1969; Scanlon, 1990). Participants in the promise condition had a choice between (a) receiving € 0.10 and doing nothing else and (b) receiving € 3, a € 0.60 stamp, and a postcard under the condition that they promised to add the € 0.60 stamp to the postcard and mail the (anonymous) postcard to a university postbox within a week of taking part in the experiment. A second group of participants also had a choice between (a) receiving € 0.10 and (b) receiving € 3, a € 0.60 stamp, and a postcard under the condition that they agreed to add the € 0.60 stamp to the postcard and mail it to a university postbox within a week of taking part in the experiment (ask condition). As in Experiment 1, participants in both groups indicated their chosen action by ticking a box. All monetary incentives were windfall money. If using the word “promise” has an effect beyond agreeing to what one was asked to do, we would expect that more participants send back the postcard in the promise than in the ask condition.

## 2. Experiment 1

### 2.1. Methods

#### 2.1.1. Participants

Data collection took place in front of a large university cafeteria on three non-successive days between January and February 2019. A total of 204 students participated with 102 students per condition (randomly assigned). The sample size was in line with our preregistered stopping rule of at least 100 participants per condition. This stopping rule accounted for slight overshooting due to participants taking part in parallel and a slight time-lag in the update of the survey count.

Participants' gender was as follows: 102–103 [50–50.05%] participants identified as female, 98–99 [48–48.5%] as male, and 2–3 [1–1.5%] preferred other gender identification.^3^ To ensure that only students participated and to prevent them from taking part twice, the last six digits of participants' student ID card were recorded (data stored separately from the study data). We asked participants to insert their card into a small cover so that only the six digits and no other information (name, photo) were visible to experimenters. Depending on their choices, students received up to € 4 for their participation.

#### 2.1.2. Procedure

We preregistered the study protocol and analyses prior to data collection on the Open Science Framework (OSF): https://osf.io/ehp3v/files/. The study was approved by the ethics committee of the Faculty of Education and Psychology, Freie Universität Berlin (approval no. 225/2018) and participants gave their informed consent before taking part in the study.

All experimenters involved in testing were blind to condition. Two experimenters recruited participants opposite the entrance to the cafeteria, using a large sign to advertise the study. A third experimenter, who did not know who was participating in the study, supervised the return box at the cafeteria's exit. The cafeteria was usually frequented by thousands of students, staff members, and visitors per day. It had an ideal layout for the study (see Supplementary Figure 1) as it featured a separate entrance and exit that were not within sight of each other. This ensured that participants could return the money out of sight of the experimenters who had recruited them. To the best of our knowledge, no security cameras were installed at the exit.

Participants completed the study on a tablet (7 inch; for detailed instructions, see Supplementary material and pre-registration), using Qualtrics. They were randomly allocated to conditions, using Qualtrics' built-in randomizer ensuring that gender was equally split by condition. After giving their informed consent, participants received a brief description of the study. Specifically, they were told that they would choose between two different amounts of money and receive the chosen amount at the end of the survey by taking a large envelope from a box. They were further told that if they took the higher amount they had a possibility to pay back money using a smaller envelope (contained in the large envelope) and drop it off at the cafeteria's exit. The instructions stressed that nobody would be able to associate the decision to pay back money with the participant and that the person supervising the return box had no information about who had participated in this study.

Participants in the promise condition were presented with the following options:

*1. You can receive* € *1 without any further consequences*.
*2. You can receive* € *4. In this case, we ask you to promise that you will pay back* € *2 when leaving the cafeteria*.

They then indicated their choice by either selecting “I choose € 1 without any further consequences.” or by selecting “I choose € 4 and I promise that I will pay back € 2.”

Participants in the control condition received the following options:

*1. You can receive* € *1 without any further consequences*.
*2. You can receive* € *4. In this case, you have the choice to pay back* € *0*, € *1*, € *2*, € *3, or* € *4 when leaving the cafeteria*.

They then either selected “I choose € 1without any further consequences.” or “I choose € 4 and I can pay back € 0, € 1, € 2, € 3, or € 4 later.”

Participants who chose € 4 in either condition received instructions to take a large envelope from a box. We had two sets of large envelopes: envelops labeled with “Q” and envelops labeled with “V” (the content was identical, apart from the letter used for labeling). The last author randomly assigned one letter to the promise and one letter to the control conditions, and experimenters remained blind to assignment throughout the study. Large envelopes featured a map of the cafeteria indicating the location of the return box (see Supplementary Figure 2). Each envelope contained four € 1 coins and a smaller return envelope. The inside of the smaller return enveloped was labeled with a small “Q” or “V” on the inside (matching the label on the larger envelope) to allow analysis of return rates per condition. Participants who chose € 1 in either condition were instructed to take an envelope labeled “X,” containing a € 1 coin, and told that they did not have to do anything else.

#### 2.1.3. Data analysis

We collected the small return envelopes and did not open them until data collection was complete to preserve experimenters' blindness to conditions (note that condition labels were inside the envelopes and not visible from the outside). We recorded how much money (and, if applicable, other content) each envelope contained. We analyzed data as preregistered. We calculated confidence intervals and performed chi-square tests and t-tests using Exploratory Software for Confidence Intervals (ESCI; Cumming, 2016). The study data can be found on OSF: https://osf.io/hkna8/.

### 2.2. Results

We first report the percentage of participants who accepted the higher payment option in each condition. In the promise condition, 77% of participants (95% *CI* = [67, 84%], *n* = 78) chose the higher amount and promised to pay back € 2 later. In the control condition, 83% of participants (95% *CI* = [75, 89%], *n* = 85) chose the higher amount. This shows that a large majority of participants in both conditions chose the higher payment option, with no significant difference between conditions [$\chi_{\left(1\right)}^{2}=1.50$, *p* = 0.221].

Next, we focused on those participants who accepted the higher payment option. In the promise condition, 63% of participants (95% *CI* = [52, 73%], *n* = 49) returned at least € 2. In the control condition, 22% of participants returned at least € 2 (95% *CI* = [15, 32%], *n* = 19). There was a significant difference between conditions in the number of people choosing to return at least € 2: $\chi_{\left(1\right)}^{2}=27.40$, *p* < 0.001.

**Figure 1:**
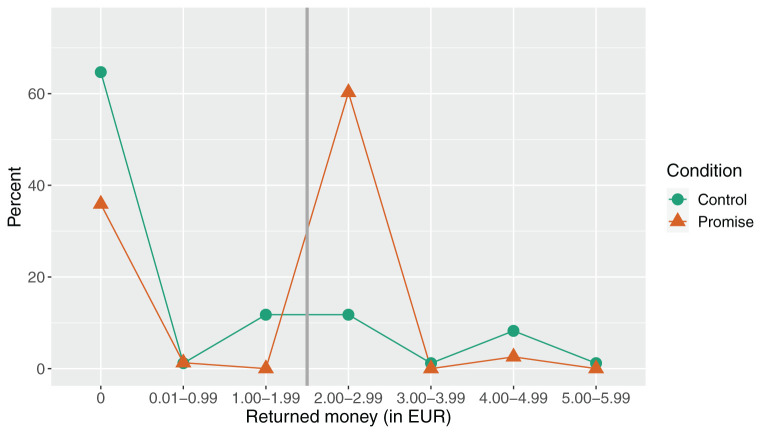
Return choices of participants in both conditions who opted for the higher payment. The x-axis shows returned amounts of money (in intervals). Participants received a score of “0” if they returned an empty envelope, returned no envelope at all, or returned other things (e.g., a 100 Kronen bill). Note that two participants in the control condition returned more than the € 4-endowment that they had received as part of the experiment. Any returned amount of € 2 or more fulfilled the promise in the promise condition (indicated by the vertical, gray line).

We also compared the average return payments. Participants in the promise condition paid back more money (*M* = € 1.31, *SD* = € 1.06, 95% *CI* = [1.07, 1.55]) than participants in the control condition (*M* = € 0.78, *SD* = € 1.33, 95% *CI* = [0.50, 1.07]). Payback differed significantly between conditions [*t*_(161)_ = 2.78, *p* = 0.006, *d* = 0.44]. For further details on the distribution of returned money see Figure 1 and Supplementary Table 1.

## 3. Experiment 2

### 3.1. Methods

#### 3.1.1. Participants

Data collection took place in front of the same university cafeteria as in Experiment 1 on six non-successive days (varying weekdays) between October and December 2019. A total of 202 students participated in the experiment with 100 in the promise and 102 in the ask condition (No data on gender were collected). We followed the pre-registered stopping rule of at least 100 participants per condition. To ensure that only students took part and that participants had not taken part in Experiment 1, we checked and recorded the last six digits of participants' student ID (in a separate file from study data). Participants received up to € 3.60 depending on their choices.

#### 3.1.2. Procedure

We preregistered the study on OSF prior to data collection: https://osf.io/u6zp3/files/. The experiment was approved by the ethics committee of the Faculty of Education and Psychology of the Freie Universität Berlin (approval no. 028/2019) and participants gave their informed consent before taking part.

Two experimenters, blind to condition, recruited participants opposite the entrance to the cafeteria. As in Experiment 1, participants completed the study on a tablet (7 inch). They were randomly assigned to conditions using Qualtrics' built-in randomizer. After giving their informed consent, they received the study instructions. They were informed that they would choose between two different amounts of money and receive the chosen amount at the end of the survey by taking an envelope from a bag. They were further told that if they chose the higher amount the envelope would also contain a stamp and a postcard with a date indicating a deadline for sending back the card (see below). The instructions stressed that the decision to send back the postcard could not be traced back to individual participants. For detailed instructions, see Supplementary material and preregistration on OSF (https://osf.io/u6zp3/files/).

In the promise condition, participants saw the following options:

*1. You can receive* € *0.10 without any further consequences*.
*2. You can receive* € *3.00 and a* € *0.60 stamp (total value:* € *3.60). In this case, we ask you to promise that you will add a* € *0.60 stamp to the enclosed postcard and send it back to us until the deadline indicated on the card*.

They then selected an option by clicking “I choose € 0.10 without any further consequences.” or “I choose € 3.00 and a € 0.60 stamp with a total value of € 3.60 and I promise that I will mail back the enclosed postcard, franked with € 0.60, until the indicated deadline (postmark).”

In the ask condition, participants were presented with the following options:

*1. You can receive* € *0.10 without any further consequences*.
*2. You can receive* € *3.00 and a* € *0.60 stamp (total value:* € *3.60). In this case, we ask you to add a* € *0.60 stamp to the enclosed postcard and send it back to us until the deadline indicated on the card*.

They indicated their choice by clicking “I choose € 0.10 without any further consequences.” or “I choose € 3.00 and a € 0.60 stamp with a total value of 3.60€ and I will mail back the enclosed postcard, franked with € 0.60, until the indicated deadline (postmark).”

Participants who chose the higher payment received envelopes marked with “Q” or “V.” As in Experiment 1, the last author randomly assigned letters to conditions and experimenters were blind to assignment. Each envelope contained three € 1 coins, a postcard, and a € 0.60 stamp to mail the postcard. The front of the postcard featured the return address (a postbox rented for the experiment), the condition code (Q/V), and a stamped return-by date (see Supplementary Figure 3). The back of the postcard featured the university logo and a field for comments (see Supplementary Figure 3). Participants who chose the lower payment amount in either condition were instructed to take an envelope labeled “X,” containing a € 0.10 coin, and told that they did not have to do anything else.

#### 3.1.3. Data analyses

Once data collection was completed, we counted returned postcards and noted whether they had arrived on time. We also recorded any comments on the cards. Cards with difficult to read postmarks were photographed and digitally enlarged to disambiguate the date on the stamp. We analyzed data as preregistered. We calculated confidence intervals and conducted chi-square tests using ESCI (Cumming, 2016). The study data is available on OSF: https://osf.io/hkna8/.

### 3.2. Results

As in Experiment 1, we first analyzed how many participants accepted the higher payment option in each condition. The majority of participants accepted the higher payment option in both the promise condition (89%, 95% *CI* = [81, 94%], *n* = 89) and the ask condition (89%, 95% *CI* = [82, 94%], *n* = 91). There was no significant difference between conditions [$\chi_{\left(1\right)}^{2}=0.002$, *p* = 0.961].

Next, we analyzed return rates of postcards for those participants who had accepted the higher payment. In line with our pre-registration, we only counted postcards as fulfilling the criterion that arrived on time with a stamp and a legible postmark. In the promise condition, 54% of postcards (95% *CI* = [44, 64%], *n* = 48) arrived on time. In the ask condition, 52% of postcards (95% *CI* = [42, 62%], *n* = 47) arrived on time. There was no significant difference between conditions [$\chi_{\left(1\right)}^{2}=0.09,p=0.759$].

In an exploratory analyses, we applied a more lenient criterion and also included postcards that arrived late or with no or ambiguous postmarks. In the promise condition, two cards arrived late, two without postmark and one with an ambiguous postmark^4^, resulting in a 60% return rate (95% *CI* = [49, 69%], *n* = 53). In the ask condition, three postcards arrived late and two cards had ambiguous postmarks, resulting in a 57% return rate (95% *CI* = [47, 67%], *n* = 52). Again, the difference between conditions was not significant [$\chi_{\left(1\right)}^{2}=0.11,p=0.743$].

Examples of comments on returned postcards can be found in the Supplementary material. We refrained from developing a coding scheme for the comments as there were not sufficient comments for systematic coding and analyses.

## 4. General discussion

Promises can be powerful devices for fostering prosocial behavior and cooperation (Ostrom et al., 1992; Bicchieri and Lev-On, 2007), but their effectiveness has been predominantly studied in lab settings. We found in two preregistered field experiments that the majority of participants (German university students) kept their word even when it was costly and effortful for them.

Our study extends previous work with US crowd-sourced workers and Indian adolescents (Woike and Kanngiesser, 2019; Kanngiesser et al., 2021) and shows that incentivized, voluntary promises are effective commitment devices. Our findings connect with recent research investigating whether participants returned a supposedly lost wallet, which found that most German participants were honest and returned the wallet (Cohn et al., 2019). In contrast to Cohn et al. (2019), our paradigm is deception-free, does not rely on covert confederates, and asks participants for their informed consent. This ensures that participants are not unknowingly burdened with participating in a study they did not consent to. Moreover, the voluntary nature of the promise ensures that participants' autonomy was respected (Hertwig and Ortmann, 2008).

Our findings from Experiment 2 revealed similar return rates in the promise and the ask condition, indicating that the wording of the commitment had little impact in our study. Analyses of communication in a high stakes TV show have found that explicit statements of commitment led to higher cooperation rates than implicit statements (Turmunkh et al., 2019). Moreover, Woike and Kanngiesser (2019) found that MTurk participants more likely to keep a costly commitment to return money when they explicitly promised as compared to being asked to do it; though payback rates in the ask condition were substantially higher than in a no-commitment control condition. Depending on context, pilot testing of wording may be needed to ensure that commitments are formulated in a way that ensures high compliance rates. Moreover, overt signals of commitment such as pins may further increase compliance (Baca-Motes et al., 2013; Kessler, 2017).

Our field study recruited German university students as participants and we chose incentives that were appropriate and proportionate for this context and population. Applications in other contexts or with other populations may require some adjustment of incentives. For example, more than 90% of US crowd-sourced workers kept their promises for (bonus) incentives as small as $0.10 and raising those incentives ten-fold from $0.20 (and a promise to pay-back $0.10) to $2.00 (and a promise to pay back $1.00) only had a small effect (Woike and Kanngiesser, 2019).

Previous studies have found cross-cultural variation in people's reasoning about promissory obligations (Miller and Bersoff, 1992; Song et al., 2009). They have also found that promises are expressed differently in different languages (Egner, 2006). Systematic cross-cultural studies on promise-keeping under incentivized conditions are still lacking and would be an interesting avenue for future research.

The study focused on investigating the effectiveness of a voluntary promise intervention under field conditions, but was not designed to test why people keep their promises. Different proposals have been made for why people keep their commitments including a preference for keeping one's word (Ellingsen and Johannesson, 2004; Vanberg, 2008), an aversion to disappointing others' expectations (Charness and Dufwenberg, 2006), or a combination of both accounts (Mischkowski et al., 2019). As decisions in our study could not be linked to individual participants (and this was stressed in the instructions), we can rule out reputational costs or fear of sanctions as potential motives. However, we are unable to say whether participants kept their word because they preferred to do so or because they thought the experimenters expected them to keep it (or both).

In summary, the majority of participants in our study kept their word at a personal cost while engaging in everyday activities even when there were no foreseeable negative consequences or reputational costs for breaking it. Our paradigm offers an easy-to-implement tool for eliciting voluntary commitments that could be leveraged, for example, to increase pro-environmental behavior (Baca-Motes et al., 2013), timely tax payments (Koessler et al., 2019), or school attendance (Benhassine et al., 2015).

## Data availability statement

The datasets presented in this study can be found in online repositories. The names of the repository/repositories and accession number(s) can be found at: https://osf.io/hkna8/.

## Ethics statement

The studies involving human participants were reviewed and approved by Ethics Committee of the Faculty of Education and Psychology, Freie Universität Berlin. The patients/participants provided their written informed consent to participate in this study.

## Author contributions

PK, DS, and JW designed the study. DS collected the data. PK analyzed the data and wrote the first draft of the manuscript. All authors contributed to manuscript revision, read, and approved the submitted version.

## Footnotes

^1^Note though that the authors also coded statements of intent such as “I will do X” as promises.

^2^Student meals in Germany are subsidized and are usually at least 50% cheaper than meal prizes for staff or the general public. At the time of the study, main meals usually cost € 1.50–€ 4 and salads, desserts, and side dishes cost < € 1 each for students.

^3^Our Qualtrics file shows 205 participants who had completed the survey [102 promise, 103 control condition], but our counting of handed out envelopes revealed that one participant in the control condition had not taken a large envelope, so that our effective sample size was 102 in the control condition. Due to the data being anonymous, we were not able to verify the gender of this participant.

^4^We excluded one card because it was returned without the promised stamp, which nevertheless seems to have been processed by the postal service.
