# Supplementary material for: Promises on the go: A field study on keeping one's word
Source: Front Psychol. 2023 Mar 6;14:1097239. doi: 10.3389/fpsyg.2023.1097239 (PMC10025327; doi:10.3389/fpsyg.2023.1097239)
Supplement: Supplementary file 1 [file Data_Sheet_1.PDF]

Supplementary Information:

Promises on the go: A field study on keeping one's word

Patricia Kanngiesser[1,2*], Daniil Serko[3], and Jan K. Woike[2,4]

[1]Faculty of Education and Psychology, Freie Universität Berlin, Berlin, Germany

[2]School of Psychology, University of Plymouth, Plymouth, UK

[3]Max Planck Research Group iSearch, Max Planck Institute for Human Development, Berlin, Germany

[4]Center for Adaptive Rationality (ARC), Max Planck Institute for Human Development, Berlin, Germany

[*]Corresponding author: patricia.kanngiesser@plymouth.ac.uk

Contents

1 Experiment 1 **1**
1.1 Set-up and materials 1
1.2 Instructions 4
1.3 Piloting 6
1.4 Results: Distribution of returned money 6

2 Experiment 2 **7**
2.1 Set-up and materials 7
2.2 Instructions 7
2.3 Piloting 9
2.4 Results 9
2.4.1 Examples of comments on postcards 9

1 Experiment 1

1.1 Set-up and materials

We conducted the experiment in front of a university cafeteria (opposite the cafeteria's entrance). The cafeteria had a separate entrance and exit (see Fig. S1), and we placed the return box inside the cafeteria, near the exit (see Fig. S1c). The location of the return box and the location of study participation were spatially separated and not within sight of each other (see Fig. S1c).

Participants who chose the higher payment option received a large envelope labelled with ”Q” or ”V” (depending on condition) and a sketch of the return location (see Fig. S2a, Fig. S2b). The large envelope contained a smaller envelope (see Fig. S2c) and four € 1 coins. Participants who chose the lower payment option received a large envelope labelled with ”X” that contained one € 1 coin.

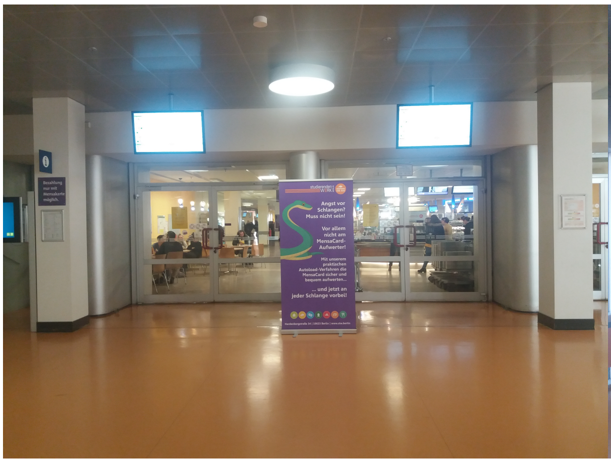

(a) entrance

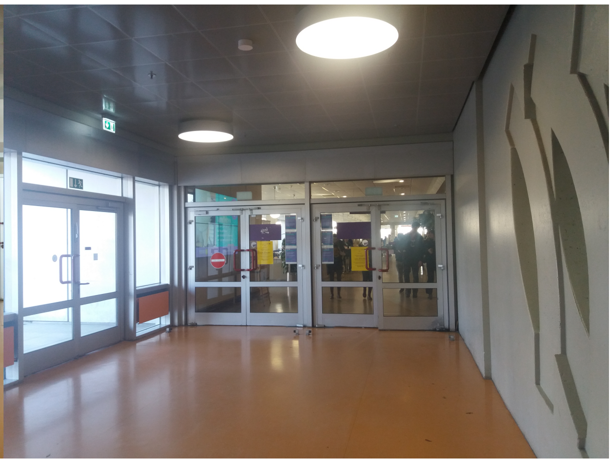

(b) exit

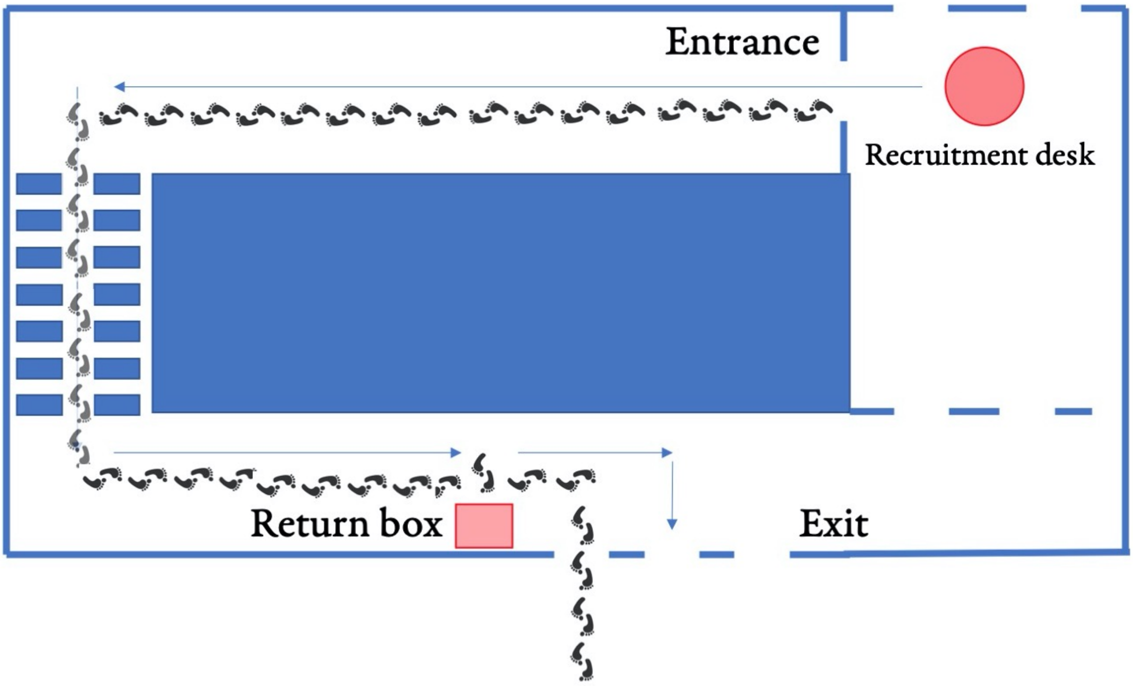

(c) schematic

Figure S1. Images of (a) the entrance to and (b) exit of the university cafeteria. The schematic (c) shows that entrance and exit were spatially separated so that the experimenters were not able to observe the return of envelopes at the cafeteria's exit.

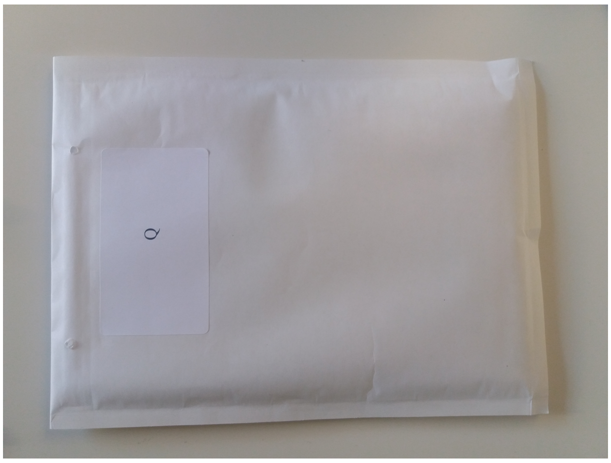

(a) front of large envelope

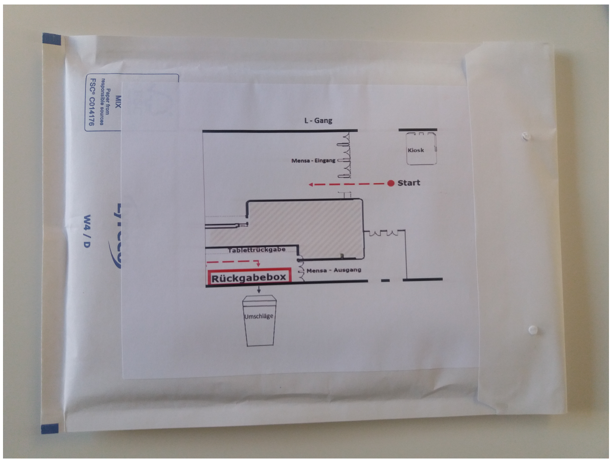

(b) back of large envelope

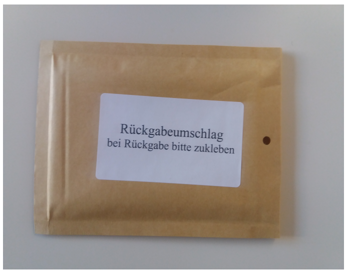

(c) smaller return envelope

Figure S2. Example of the front (a) and back (b) of a large envelope that participants received who chose the higher payment option. On the back of the large envelope a map indicated the location of the return box. The large envelope contained four € 1 coins and a smaller return envelope (c).

1.2 Instructions

Note that the following instructions are a translation from the German instructions. For German instructions, see the pre-registration on the Open Science Framework: https://osf.io/hkna8/.

After giving their informed consent and entering their gender, participants read the following instructions:

Page 1: All conditions

Instructions

Please carefully read the following text.

On the following pages, you will have the choice between different amounts of money. You will receive the selected amount of money at the end of the survey by taking an envelope from the box indicated on the screen. If you decide to take the higher amount, you will have the possibility to pay back money. For this, you will find a smaller, yellow envelope in your envelope in addition to the money. You can return this smaller, yellow envelope by placing it in a labelled box at the exit of the cafeteria (opposite the tray return). The box is labelled ”envelopes”.

The decision to pay back money cannot be associated with your person by the experimenters. The person supervising the box at the exit has no information about who has participated in this study.

If you have understood these instructions, please click on the arrow. If you have any questions, please contact the experimenters.

Page 2: Promise condition

You have two choices:

1. You can receive € 1 without any further consequences.
2. You can receive € 4. In this case, we ask you to promise that you will pay back € 2 when leaving the cafeteria.

○ 1. I choose € 1 without any further consequences.

○ 2. I choose € 4 and I promise that I will pay back € 2.

Page 2: Control condition

You have two choices:

1. You can receive € 1 without any further consequences.
2. You can receive € 4. In this case, you have the choice to pay back € 0, € 1, € 2, € 3, or € 4 when leaving the cafeteria.

○ 1. I choose € 1without any further consequences.

○ 2. I choose € 4 and I can pay back € 0, € 1, € 2, € 3, or € 4 later.

Page 3: Higher payment (all conditions)

In a moment, please take an envelope out of box ”V”/”Q”.
The envelope contains € 4 and a small, yellow envelope (see image). If you want to pay back money, please place the chosen amount of money in the small, yellow envelope and close it.

Small, yellow envelope to return money

image of small envelope

Please place the closed envelope into the box labelled ”envelopes” at the exit of the cafeteria (opposite the tray return; see images).
If you do not want to return any money, you do not have to return the envelope.

Return box for yellow envelopes opposite tray return.

image of return box, schematic of cafeteria & return location

The decision to return money cannot be associated with your person by the experimenters and the envelopes cannot be tracked by us. The person supervising the return box does not communicate with the experimenters at the entrance and has no information about whether you have participated in the study.

Page 4: Higher payment (all conditions)

Please return the tablet to the experimenters and take an envelope from box **"V"/"Q"**.

This terminates the survey.

Thank you for your participation!

Page 3: Lower payment (all conditions)

Please return the tablet to the experimenters and take an envelope from box **"X"**. The envelope contains € 1 that you may keep. You do not have to do anything else. This terminates the study for you.

Thank you for your participation!

1.3 Piloting

We piloted the study with 21 additional participants. Piloting resulted in small adjustments to wording to remove ambiguities in study instructions. We also added a map to the envelopes to help participants find the location of the return box. We pre-registered the study after piloting and prior to the main data collection.

1.4 Results: Distribution of returned money

Table S1. Overview distribution of content of returned envelopes

Condition	Empty	100 Kronen	€ 0.01	€ 0.13	€ 1.00	€ 2.00	€ 3.00	€ 4.00	€ 4.50	€ 5.00
Control	18	1	1	0	10	10	1	6	1	1
Promise	5	0	0	1	0	47	0	2	0	0

2 Experiment 2

2.1 Set-up and materials

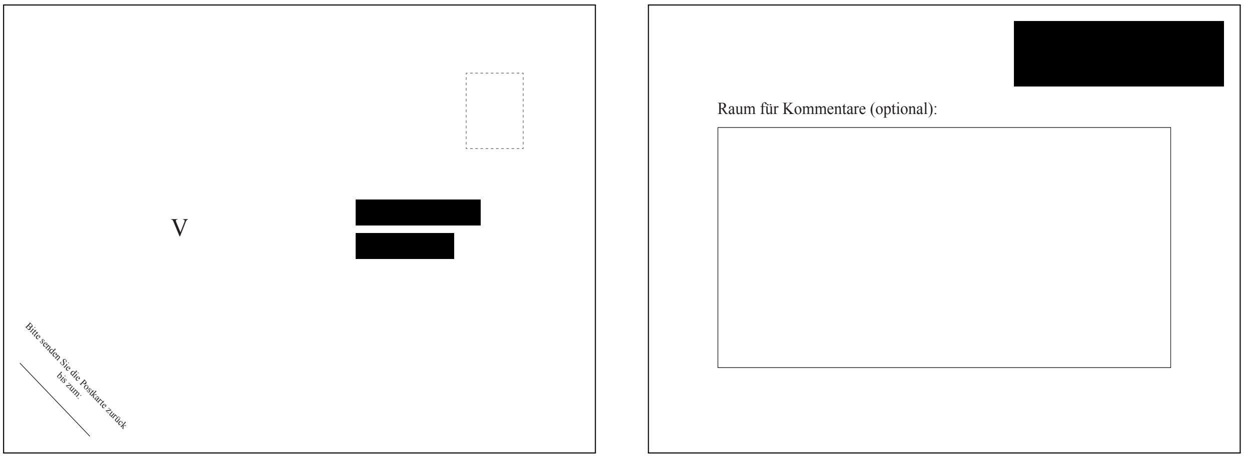

(a) postcard front **(b)** postcard back

Figure S3. Example of postcard used in the experiment. The front (a) displayed the return date (left bottom corner), the postbox address, and a letter (”Q”/”V”) indicating condition. The back (b) displayed a section for comments and the university logo (top right corner). Postbox address and university logo are redacted.

2.2 Instructions

Note that the following instructions are a translation from the German instructions. For the original German instructions, see the pre-registration:

https://osf.io/hkna8/?view_only=fc89b70bb37147ea869598844d80e9f0.

After giving their informed consent participants read the following instructions:

Page 1: All conditions

Instructions

Please read the following text carefully.

On the following pages, you will make a single choice between different amounts of money. You will receive the selected amount of money by taking an envelope out of a bag indicated on the screen. If you decide to take the higher amount, there will also be a postcard and a stamp in the envelope. You can

send this postcard back to us (until the date indicated on the card).

The decision to send back the postcard cannot be associated with your person. We do not save any information about who has participated in the study (other than the list with the six-digit code).

If you have understood these instructions, please click on the arrow. If you have any questions, please contact the experimenters.

Page 2: Promise condition

You have two choices (this is the only question in this survey):

1. You can receive € 0.10 without any further consequences.
2. You can receive € 3.00 and a € 0.60 stamp (total value: € 3.60). In this case, we ask you to promise that you will add a € 0.60 stamp to the enclosed postcard and send it back to us until the deadline indicated on the card.

○ 1. I choose € 0.10 without any further consequences.

○ 2. I choose € 3.00 and a € 0.60 stamp with a total value of € 3.60 and I promise that I will mail back the enclosed postcard, franked with € 0.60, until the indicated deadline (postmark).

Page 2: Ask condition

You have two choices (this is the only question in this survey):

1. You can receive € 0.10 without any further consequences.
2. You can receive € 3.00 and a € 0.60 stamp (total value: € 3.60). In this case, we ask you to add a € 0.60 stamp to the enclosed postcard and send it back to us until the deadline indicated on the card.

○ 1. I choose € 0.10 without any further consequences.

○ 2. I choose € 3.00 and a € 0.60 stamp with a total value of 3.60€ and I will mail back the enclosed postcard, franked with € 0.60, until the indicated deadline (postmark).

Page 3: Higher payment (all conditions)

In a moment, please take an envelope out of bag **”V”/”Q”**.

The envelope contains € 3.00, a € 0.60 stamp (total value € 3.60) and a postcard. If you want to send the

postcard back to us, please add the € 0.60 stamp. Please make sure to send back the postcard until the deadline indicated on the card (post mark).

Your decision to send back the postcard cannot be associated with your person.

Please click on the arrow below one more time.

2.3 Piloting

We initially piloted the study in a public park in Berlin. Participants had a choice between (a) one € 0.10 stamp or (b) six € 0.60 stamps under the condition that they promised to send back a postcard (promise condition). The ask condition included the same choice but without the promise (participants were asked to send back the postcard). However, when approached by the experimenters, many people in the park showed little interest in participation. Only 30 people responded and only 12 of these (40%) opted for the higher payment across conditions. As a consequence, we decided to change the higher payment option to € 3.00 in € 1-coins and one € 0.60 stamp (instead of six € 0.60 stamps). We piloted the new incentive scheme with new participants in the park, but again people showed little interest to participate. Of those who did participate (n = 20), 9 of 11 (82%) chose the higher payment option in the promise condition, but only 1 of 9 (11%) in the ask condition. In order to be able to compare return rates of postcards across conditions, we required similar acceptance rates of higher payment across conditions. Hence, we decided to conduct the study at the same university cafeteria as in Experiment 1. To ensure that incentives and wording were appropriate, we piloted the experiment with 30 students in front of the cafeteria (data not reported in the study). We pre-registered the study after piloting and prior to the main data collection.

2.4 Results

2.4.1 Examples of comments on postcards

The majority of participants used the comment field on the post card to provide comments or to include drawings (N = 57 postcards with comments/drawings). Many participants expressed their gratitude for the received money, e.g. ”Thank you for sponsoring my coffee break.”, or expressed interest in the study design or results, ”Very exciting study design; I would love to know what the outcome is. [...]”. Several other participants commented on their motivation to return the postcard, e.g. ”Please note: I am privileged and financially supported by my parents, so I am not dependent on scamming a stamp.”
